# Оценка качества жизни пациентов после паратиреоидэктомии из стандартного и малого доступов

**DOI:** 10.14341/probl12735

**Published:** 2021-02-25

**Authors:** А. В. Огородников, С. С. Харнас

**Affiliations:** Московский научно-исследовательский онкологический институт им. П.А. Герцена, филиал Национального медицинского исследовательского центра радиологии; Университетская клиническая больница №1 Первого Московского государственного медицинского университета им. И.М. Сеченова (Сеченовский университет)

**Keywords:** первичный гиперпаратиреоз, доступ по Кохеру, малый доступ, паратиреоидэктомия, качество жизни, опросник SF-36, ЛАШ

## Abstract

ОБОСНОВАНИЕ. Первичный гиперпаратиреоз (ПГПТ) — это повышение секреторной активности околощитовидных желез (ОЩЖ) вследствие их опухолевого или гиперпластического изменения. Ввиду отсутствия эффективной альтернативы лечению ПГПТ хирургический способ по-прежнему остается единственным верным тактическим решением ведения больных с установленным диагнозом ПГПТ. В работе представлены отдаленные результаты и оценка качества жизни больных, перенесших хирургическое лечение из стандартного и малого доступов. Полученные результаты показали перспективность щадящего подхода к лечению ПГПТ, обусловленного аденомой ОЩЖ.ЦЕЛЬ. Изучение эффективности хирургического лечения ПГПТ на основании оценки качества жизни больных, перенесших паратиреоидэктомию из стандартного и малого доступов.МАТЕРИАЛЫ И МЕТОДЫ. Проведено ретроспективное исследование качества жизни пациентов с ПГПТ после хирургического лечения с использованием опросника SF-36 и линейной аналоговой шкалы (ЛАШ). Статистическая обработка данных выполнена на языке программирования R с использованием пакета FMSB. Количественные параметры были представлены в виде медианы (Меdian) и интерквартильного размаха (25-й (1st Qu) — нижний квартиль и 75-й (3rd Qu) — верхний квартиль). В качестве непараметрического статистического критерия использован U-критерий Манна–Уитни (Mann–Whitney U-test), на основании которого произведен расчет p-value. Расчетные данные результатов исследования представлены в графическом виде — в виде столбчатых диаграмм, spider plot и barplot.РЕЗУЛЬТАТЫ. В настоящем исследовании приняли участие 264 пациента. Пациенты были разделены на 2 группы: ГР1 — пациенты, оперированные из доступа по Кохеру с обязательной ревизией всех 4 ОЩЖ, ГР2 — пациенты, получившие оперативное лечение из малого доступа с удалением измененной ОЩЖ, без ревизии остальных ОЩЖ. При проведении анализа качества жизни пациентов до операции статистически значимых различий в группах по показателю PF (Physical Functioning) и VT (Vitality) выявлено не было. Проведение паратиреоидэктомии из малого доступа (пациенты ГР2) статистически значимо повышало качество жизни по домену GH (General Health) и VT (Vitality). Анализ ЛАШ до операции между группами не показал статистически значимых различий, в то время как после хирургического лечения показатели по ЛАШ отличаются в сторону улучшения в ГР2.ЗАКЛЮЧЕНИЕ. Результаты, полученные в ходе исследования, показали перспективность щадящего подхода к лечению ПГПТ, обусловленного аденомой ОЩЖ, что отражено в более высоких показателях качества жизни.

## Abstract (en)

BACKGROUND. Primary hyperparathyroidism (PGPT) is an increase in the secretory activity of the parathyroid glands (OSH), due to their tumor or hyperplastic changes. Due to the lack of an effective alternative to the treatment of PGPT, the surgical method is still the only correct tactical solution for the management of patients with an established diagnosis of PGPT. The paper presents the long-term results and assessment of the quality of life of patients who underwent surgical treatment from standard and small access. The results obtained showed the promise of a sparing approach to the treatment of PGPT caused by LV adenoma.AIM. To study the effectiveness of surgical treatment of patients with PGPT based on the assessment of the quality of life of patients who underwent parathyroidectomy from standard and small access.MATERIALS AND METHODS. A retrospective study of the quality of life of patients with PGPT after surgical treatment was conducted using the SF-36 questionnaire and the linear analog scale (LAS). Statistical data processing is performed in the R programming language using the FMSB package. The quantitative parameters were presented as median (Median) and interquartile range (25th (1st Qu) — lower quartile and 75th (3rd Qu ) — upper quartile). As a nonparametric statistical criterion, the Mann–Whitney U-test was used, on the basis of which the p-value was calculated. The calculated data of the research results are presented in graphical form — in the form of bar charts, spider plot and barplot.RESULTS. This study involved 264 patients. The patients were divided into 2 groups: GR1 — patients operated from the Kocher access with mandatory revision of all 4 OSH, GR2 — patients who received surgical treatment from the small access with the removal of the altered OSH, without revision of the remaining OSH. When analyzing the quality of life of patients before surgery, there were no statistically significant differences in the groups in terms of PF (Physical Functioning) and VT (Vitality). Small-access parathyroidectomy (patients with GR 2) significantly improved the quality of life in the GH (General Health) and VT (Vitality) domains. The analysis of LAS before surgery between the groups showed no statistically significant differences, while after surgical treatment, the indicators on the linear analog scale differ in the direction of improvement in GR2.CONCLUSION. The results obtained in the course of the study showed the promise of a gentle approach to the treatment of PGPT caused by LV adenoma, which is reflected in higher quality of life indicators.

## ОБОСНОВАНИЕ

Первичный гиперпаратиреоз (ПГПТ) — это повышение секреторной активности околощитовидных желез (ОЩЖ) вследствие их опухолевого или гиперпластического изменения. При этом на сегодняшний день ПГПТ во всем мире рассматривается как третья эндокринная эпидемия, наравне с сахарным диабетом и другими заболеваниями щитовидной железы [1][2]. Стоит отметить, что истинная распространенность этого заболевания претерпела изменения после внедрения лабораторно-диагностического алгоритма, включающего обязательное исследование общего и ионизированного кальция в стандартном биохимическом анализе крови. Ввиду отсутствия эффективной альтернативы лечению ПГПТ хирургический способ по-прежнему остается единственным верным тактическим решением ведения больных с установленным диагнозом ПГПТ [3][4]. Эффективность хирургического лечения составляет 95–98% при частоте возможных послеоперационных осложнений до 1–2% при условии высокой квалификации и опыта хирурга [5]. В связи со сложностью топической диагностики для выполнения адекватной паратиреоидэктомии, как правило, выполняется ревизия всех ОЩЖ с последующим удалением одной или нескольких пораженных желез [6–8]. Однако двусторонняя ревизия шеи ввиду травматичности приводит к таким возможным осложнениям, как травматизация возвратных гортанных нервов и сосудов шеи, проявляющаяся парезом и кровотечением соответственно.

Наряду с этим появляются сообщения о возможности использования малого доступа при удалении аденомы ОЩЖ без ревизии остальных ОЩЖ. Сторонники паратиреоидэктомии из малого доступа считают, что это позволяет уменьшить продолжительность госпитализации, улучшить течение послеоперационного периода и косметические результаты [9]. В 1987 г. Д.Н. Нурманбетовым были рассмотрены основные принципы определения исхода хирургического лечения больных с ПГПТ. Исследование отдаленных результатов осуществлялось в период от 6 мес до 10 лет с момента операции. Как правило, при успешном проведении лечения в течение первого года исчезают проявления ПГПТ как субъективного, так и объективного характера, нормализуются лабораторные показатели уровня кальция в сыворотке крови. При удовлетворительном результате хирургического лечения также отмечается нормальный уровень кальция, однако у пациента остаются выраженные жалобы, в результате чего отмечается снижение работоспособности. Неудовлетворительный же результат лечения приводит к сохранению гиперкальциемии, дальнейшему проявлению почечных и костных изменений. Правомочность и целесообразность экономных операций при ПГПТ могут быть оценены только на основании тщательного анализа отдаленных результатов операций, чему и посвящено настоящее исследование.

Качество жизни является суммарной характеристикой физического, психического и социального функционирования человека, в основе которой лежит его субъективное восприятие состояния здоровья. Оценка качества жизни является важным критерием эффективности проведенного хирургического лечения, позволяющим точно описать состояние пациента до операции и в момент его реабилитации [10].

## ЦЕЛЬ ИССЛЕДОВАНИЯ:

## МАТЕРИАЛЫ И МЕТОДЫ

## Место и время проведения исследования

Проведено ретроспективное исследование качества жизни пациентов с ПГПТ, находившихся на хирургическом лечении в клинике факультетской хирургии №1 им. Н.Н. Бурденко на базе УКБ №1 ФГАОУ ВО «Первый Московский государственный медицинский университет им. И.М. Сеченова» Министерства здравоохранения РФ с 2009 г. по 2017 г.

## Методы

Проведена оценка качества жизни пациентов с использованием опросника SF-36 и линейной аналоговой шкалы (ЛАШ) до операции (за 2 дня) и в отдаленном периоде (в среднем 31±5 мес).

Опросник SF-36 (SF-36 Healht Status Survey) представляет собой неспецифический опросник для оценки качества жизни, особенно широко распространенный в США и странах Европы, где впервые были проведены исследования отдельных популяций и получены результаты по нормам для здорового населения и для групп больных с различными заболеваниями. Опросник отражает не только общее благополучие, но и степень удовлетворенности теми сторонами жизнедеятельности человека, на которые влияет состояние его здоровья. SF-36 состоит из 36 вопросов, которые сгруппированы в восемь шкал.

Результаты были представлены в виде балльной оценки по 8 шкалам, включающим следующие домены:

Более высокий показатель по шкале (от 0 до 100) соответствовал более высокому показателю качества жизни. Затем шкалы формировались в два показателя, отражающие «физический компонент здоровья» (PH) и «психологический компонент здоровья» (MH).

В заключительной части исследования отдаленных результатов лечения использовалась ЛАШ как показатель самостоятельной оценки последствий операции пациентом на основании измерения его функционального статуса и общего состояния здоровья. Пациентам предоставлялась ЛАШ, на которой они самостоятельно отмечали вертикальной чертой общее состояние своего здоровья от «очень плохого» к «очень хорошему».

## Статистический анализ

Статистическая обработка данных была выполнена на языке программирования R с использованием пакета FMSB. Количественные параметры были представлены в виде медианы (Меdian) и интерквартильного размаха (1st Qu — нижний квартиль и 3rd Qu — верхний квартиль). В качестве непараметрического статистического критерия использован U-критерий Манна–Уитни (Mann–Whitney U-test), на основании которого произведен расчет p-value. Расчетные данные результатов исследования представлены в графическом виде — в виде столбчатых диаграмм, spider plot и barplot.

## Этическая экспертиза

Исследование одобрено Локальным этическим комитетом ФГАОУ ВО «ПМГМУ им. И.М. Сеченова» Минздрава России (Сеченовский Университет) (выписка из протокола №06-21 от 07.04.2021).

## РЕЗУЛЬТАТЫ

В настоящем исследовании приняли участие 264 пациента. Пациенты были разделены на 2 группы по объему и типу доступа оперативного вмешательства:

Первоначально был проведен анализ «сырых» данных на нормальность, от чего в дальнейшем зависела возможность использования параметрических статистических тестов и проведение оценки среднего (рис. 1, 2, 3, 4).

**Figure:**
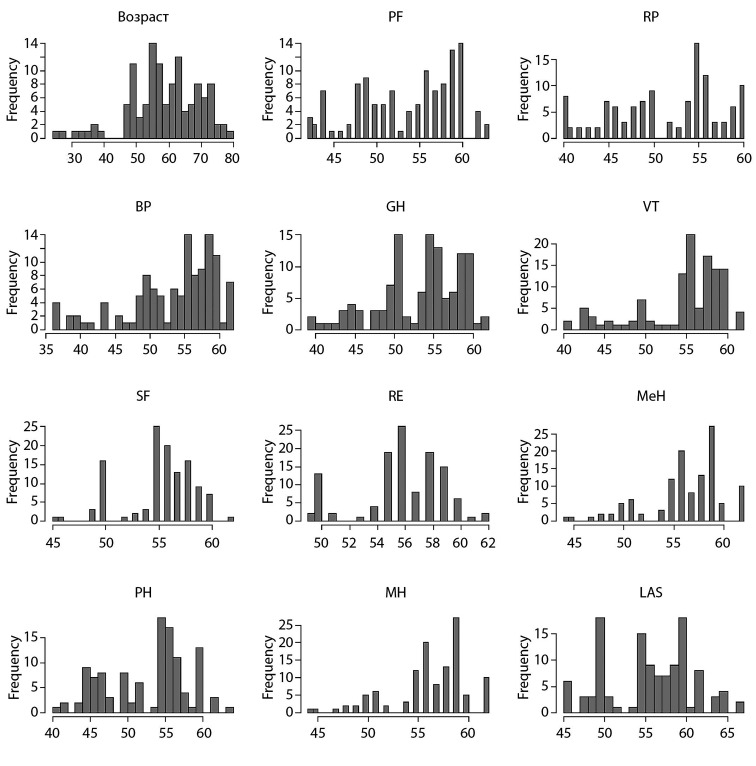
Рисунок 1. Распределение данных пациентов 1-й группы по шкале SF-36 до операции.

**Figure:**
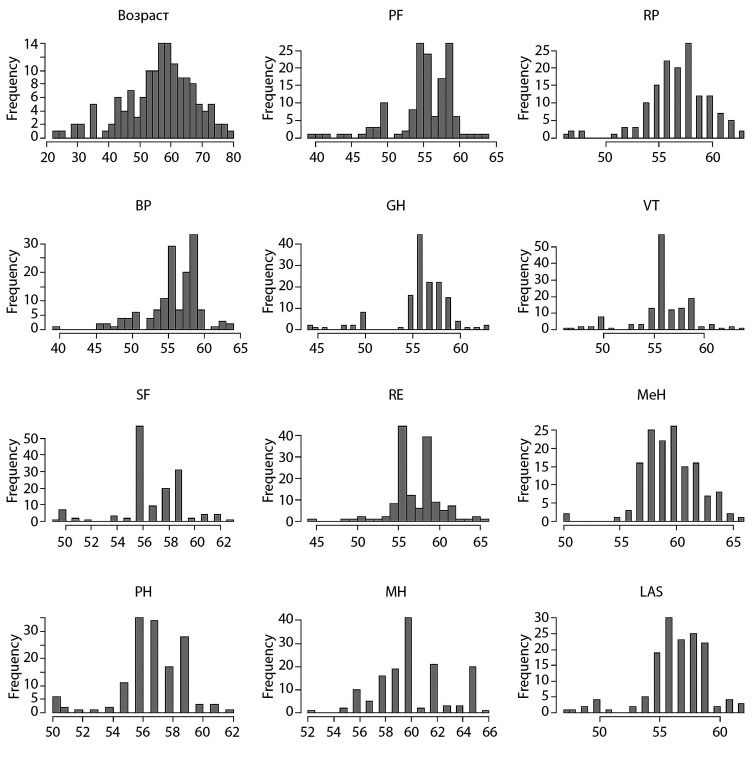
Рисунок 2. Распределение данных пациентов 2-й группы по шкале SF-36 до операции.

**Figure:**
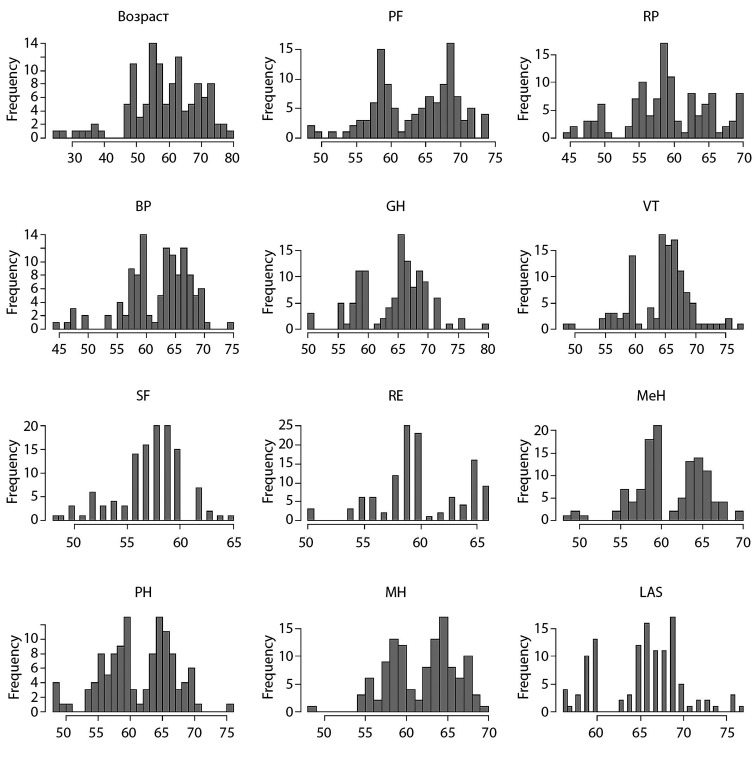
Рисунок 3. Распределение данных пациентов 1-й группы по шкале SF-36 после операции.

**Figure:**
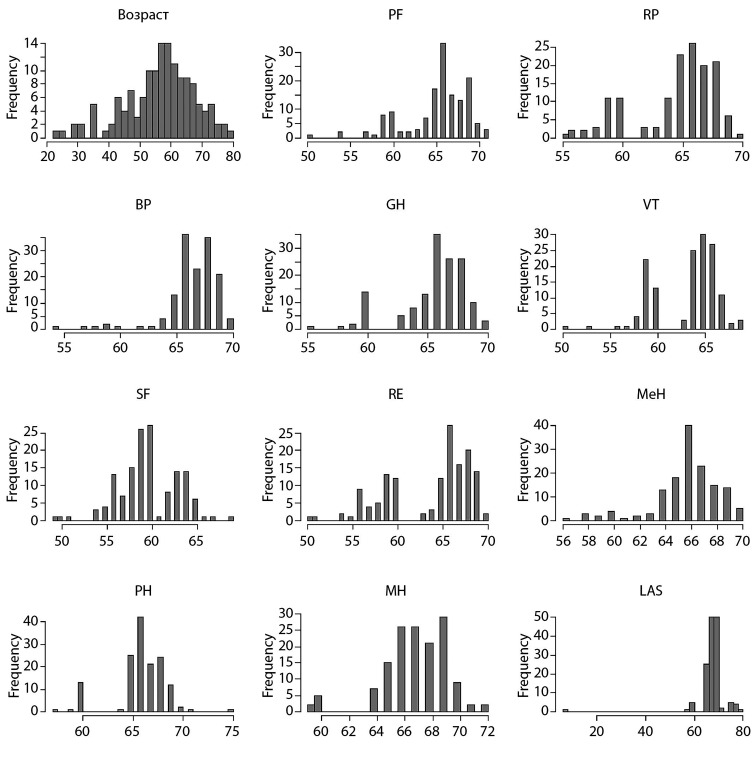
Рисунок 4. Распределение данных пациентов 2-й группы по шкале SF-36 после операции.

Исходя из этого, мы выяснили, что распределение данных ненормальное. По этой причине в настоящем исследовании оценивали медиану (Меdian), а не среднее значение. Также были использованы непараметрические тесты при определении уровня значимости в отличиях — в нашем случае для парного сравнения использовался критерий Манна–Уитни.

С помощью критерия Манна–Уитни была проведена проверка статистической значимости отличий в различных параметрах для разных групп. Показатели, p-уровень значимости ниже стандартной установленной границы (0,05) отмечены светло-серым (табл. 1).

Так как параметры рассматриваются и сравниваются независимо, в данном случае поправка на множественное сравнение не требуется.

Таким образом, при проведении анализа качества жизни пациентов до операции статистически значимых различий в группах по показателям PF (Physical Functioning) и VT (Vitality) выявлено не было.

При сравнении групп после операции возникает большее число статистически значимых отличий (табл. 2). В обеих группах хирургическое лечение приводило к достоверному улучшению качества жизни по всем показателям. Проведение паратиреоидэктомии из малого доступа (пациенты ГР2) статистически значимо повышало качество жизни по доменам GH (General Health) и VT (Vitality) в сравнении с пациентами ГР1.

**Table:** Таблица 1. Показатели качества жизни по двум группам пациентов до операции по опроснику SF-36

	Группа 1	Группа 2	pval
	Min.	1st Qu.	Median	Mean	3rd Qu.	Max.	Min.	1st Qu.	Median	Mean	3rd Qu.	Max.
Возраст	24	53	59,5	59,24	67	79	23	52	58	56,94	64,25	79	1,17E-01
PF	42	49	55	53,79	59	63	39	55	56	55,55	59	64	6,88E-02
RP	40	47	53,5	51,47	56	60	46	55	57	56,92	59	63	8,57E-15
BP	36	50	56	53,87	59	62	39	55	57	56,24	59	64	2,13E-02
GH	39	51	55	53,74	58	62	44	56	56	56,08	58	63	3,74E-04
VT	40	55	56	55,08	59	62	46	56	56	56,11	58	64	9,63E-01
SF	45	55	56	55,38	58	62	49	56	56	56,9	59	63	3,79E-05
RE	49	55	56	56	58	62	44	56	57	57,62	59	66	2,05E-05
MeH	44	55	57	56,4	59	62	50	58	60	59,72	61	66	2,10E-14
PH	40	48	55	53,15	57	64	50	56	57	56,81	58	62	1,63E-09
MH	44	55	57	56,4	59	62	52	59	60	60,33	62	66	4,97E-18
LAS	38	43,25	49,5	49,02	53	60	42	55	52	51,62	53	57	8,49E-01

В данной таблице для каждого признака для пациентов двух групп до операции указаны следующие статистические характеристики: Min. — минимальное значение данного параметра в данной группе; 1st Qu. — граница первого (нижнего) квартиля (25 процентов значений); Median — медиана; Mean — среднее арифметическое; 3rd Qu. — граница третьего (верхнего) квартиля (75 процентов значений); Max. — максимальное значение данного параметра в данной группе.

**Table:** Таблица 2. Показатели качества жизни по двум группам пациентов после операции по опроснику SF-36

	Группа 1	Группа 2	pval
	Min.	1st Qu.	Median	Mean	3rd Qu.	Max.	Min.	1st Qu.	Median	Mean	3rd Qu.	Max.
Возраст	24	53	59,5	59,24	67	79	23	52	58	56,94	64,25	79	1,17E-01
PF	48	59	65	63,99	69	74	50	64	66	65,34	68	71	1,97E-01
RP	44	56	59	59,5	64	70	55	63,75	66	64,62	67	70	4,30E-13
BP	44	59	64	62,58	67	75	54	66	67	66,66	68	70	6,44E-12
GH	50	60	66	64,84	68,75	80	55	65	66	65,72	68	70	4,37E-01
VT	48	61,5	66	64,93	68	78	50	60	65	63,38	66	69	3,58E-05
SF	48	56	58	57,42	59	65	49	58	60	59,79	63	69	2,62E-08
RE	50	58	60	60,14	63	66	50	59,75	66	63,83	67,25	70	1,08E-11
MeH	48	59	60	61,39	65	70	56	65	66	65,84	67	70	4,65E-19
PH	48	58	61,5	61,74	66	76	57	65	66	66,04	68	75	1,04E-12
MH	48	59	63	62,44	65	70	59	66	67	66,97	69	72	7,71E-21
LAS	64	68	74	73,48	77	85	73	82	83	82,88	84,25	95	2,44E-07

Эти же отличия хорошо заметны на spider plot (рис. 5, 6, 7).

**Figure:**
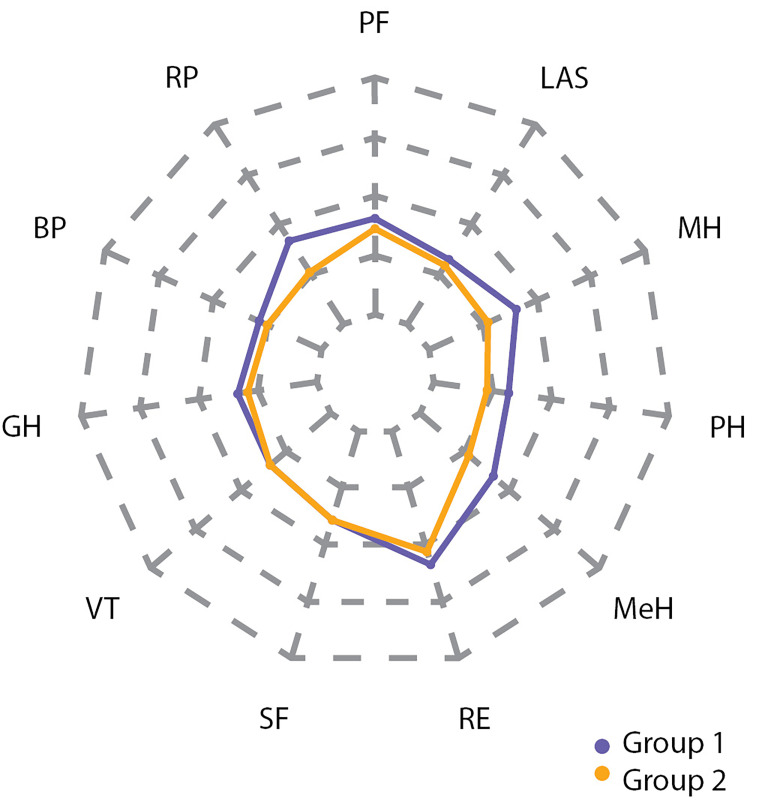
Рисунок 5. Разница в показателях между группами 1 и 2 по шкалам SF-36 до операции.

**Figure:**
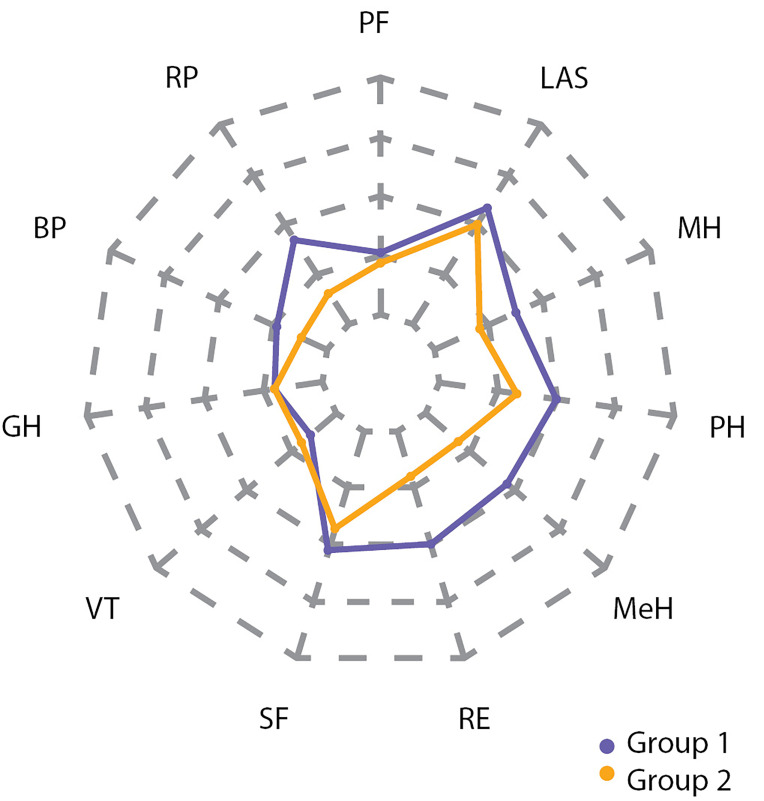
Рисунок 6. Разница в показателях между группами 1 и 2 по шкалам SF-36 после операции.

**Figure:**
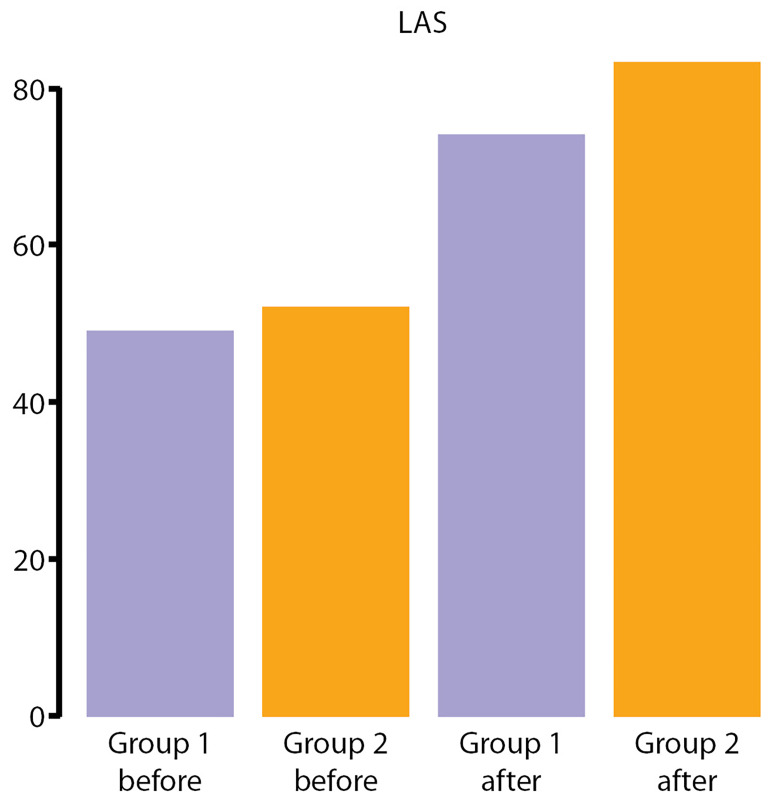
Рисунок 7. Визуализация линейной аналоговой шкалы в 1-й и 2-й группах до и после операции.

Для анализа результатов ЛАШ использовались значения медианы (Median). Исходя из полученных данных, можно сделать однозначный вывод, что в группах до операции различия не были значимы статистически, однако после операции показатели по ЛАШ отличались в сторону улучшения в ГР2 (рис. 7).

## ОБСУЖДЕНИЕ

По результатам ряда исследований, хирургическое лечение больных с ПГПТ приводит к улучшению качества жизни пациентов, независимо от объема оперативного пособия [11–15]. Однако сторонники паратиреоидэктомии из «малого» доступа считают, что такой щадящий подход позволяет уменьшить продолжительность госпитализации, улучшить течение послеоперационного периода и косметический эффект [9]. Результаты, полученные в ходе настоящего исследования, отражают современные тенденции к преимущественному применению органосохраняющих хирургических вмешательств и соответствуют результатам отечественных и зарубежных авторов [16][17], что показывает их перспективность, проявляющуюся в более высоких показателях качества жизни.

## Клиническая значимость результатов

Хирургическое лечение пациентов с ПГПТ значительно улучшает качество их жизни после операции. Оценить результаты в отдаленный период после лечения возможно с использованием разных видов опросников, с помощью которых можно надежно проследить все закономерности проявления заболевания и нарушений определенных аспектов жизни до и после проведенного лечения. Изучение новых видов опросников имеет огромную практическую значимость для совершенствования отечественной системы медицинской помощи данной категории пациентов.

## ЗАКЛЮЧЕНИЕ

Проведение хирургического лечения пациентов с ПГПТ статистически значимо повысило качество жизни в обеих группах, при этом качество жизни пациентов после паратиреоидэктомии из малого доступа оказалось выше, чем после паратиреоидэктомии из стандартного доступа по всем показателям, однако достоверное отличие отмечено по показателям GH (General Health) и VT (Vitality). Показатель ЛАШ пациентов, перенесших паратиреоидэктомию из малого доступа, достоверно выше в сравнении с ЛАШ пациентов после паратиреоидэктомии из стандартного доступа по Кохеру.
